# State-of-the art review: Noncompaction cardiomyopathy in pediatric patients

**DOI:** 10.1007/s10741-021-10089-7

**Published:** 2021-03-14

**Authors:** Sofie Rohde, Rahatullah Muslem, Emrah Kaya, Michel Dalinghaus, Jaap I. van Waning, Danielle Majoor-Krakauer, Jeffery Towbin, Kadir Caliskan

**Affiliations:** 1 Thoraxcenter, Department of Cardiology, Erasmus University Medical Center, Room RG 431, 3015 GD Rotterdam, The Netherlands; 2 Division of Pediatric Cardiology, Sophia Children’s Hospital, Erasmus University Medical Center Rotterdam, Rotterdam, The Netherlands; 3 Department of Clinical Genetics, Erasmus University Medical Center, Rotterdam, The Netherlands; 4 The Heart Institute, Le Bonheur Children’s Hospital, Memphis, TN USA

**Keywords:** Noncompaction cardiomyopathy, Genetics, Heart failure, Pediatric, Children, Review

## Abstract

Noncompaction cardiomyopathy (NCCM) is a disease characterized by hypertrabeculation, commonly hypothesized due to an arrest in compaction during fetal development. In 2006, NCCM was classified as a distinct form of cardiomyopathy (CMP) by the American Heart Association. NCCM in childhood is more frequently familial than when diagnosed in adulthood and is associated with other congenital heart diseases (CHDs), other genetic CMPs, and neuromuscular diseases (NMDs). It is yet a rare cardiac diseased with an estimated incidence of 0.12 per 100.000 in children up to 10 years of age. Diagnosing NCCM can be challenging due to non-uniform diagnostic criteria, unawareness, presumed other CMPs, and presence of CHD. Therefore, the incidence of NCCM in children might be an underestimation. Nonetheless, NCCM is the third most common cardiomyopathy in childhood and is associated with heart failure, arrhythmias, and/or thromboembolic events. This state-of-the-art review provides an overview on pediatric NCCM. In addition, we discuss the natural history, epidemiology, genetics, clinical presentation, outcome, and therapeutic options of NCCM in pediatric patients, including fetuses, neonates, infants, and children. Furthermore, we provide a simple classification of different forms of the disease. Finally, the differences between the pediatric population and the adult population are described.

## Introduction

Noncompaction cardiomyopathy (NCCM) is heterogenous cardiomyopathy characterized by multiple prominent trabeculations and deep intertrabecular recesses. It is a relatively new, rare disease entity that has only been recognized as a distinct cardiomyopathy since 2006 [1].

Since its first description in 1984 by Engberding et al. [2], NCCM has been labeled with several names over the last 35 years including spongy myocardium, left ventricular noncompaction cardiomyopathy (LVNC), and left ventricular hypertrabeculation (LVHT) [3, 4]. In this paper, we will use NCCM as the main term to address this disease, in analogy to the terminology of dilated cardiomyopathy (DCM), hypertrophic cardiomyopathy (HCM), and restrictive cardiomyopathies (RCM) and given the involvement of right ventricular involvement in many patients [5, 6].

Although NCCM is a rare cardiac disease, it is the third most common cardiomyopathy (CMP) in the pediatrics following DCM and HCM [7]. NCCM can cause heart failure and is associated with thromboembolic events and (fatal) arrhythmias in adults [8]. In addition, NCCM can occur as an isolated or non-isolated phenotype. Non-isolated NCCM may be accompanied by congenital heart diseases (CHDs), features of other CMPs, and/or neuromuscular diseases (NMDs) [9, 10].

The pathophysiology of NCCM is not completely understood and there are different theories of how the extensive trabeculations are formed. One of those is that NCCM is caused by abnormal embryological compaction of the myocardium, leading to a hypertrabeculated honeycomb-like myocardium [11, 12]. However, this does not explain NCCM diagnosed in adulthood. Many genes have been reported to be associated with NCCM, but none of the proposed pathogenic gene variants or chromosomal defects can directly be linked to a disrupted compaction process in the fetus. Furthermore, most of the (likely) pathogenic gene variants can lead to different phenotypes [13].

Due to its rarity, the lack of universally accepted diagnostic criteria, and the lack of awareness among the clinicians, little is known about NCCM in children. In particular, very few cases of fetal NCCM diagnosis have been reported [14, 15]. Among those are cases caused by pathogenic MYH7 gene variant. These observations do not support or explain abnormal myocardial maturation during gestation. Genetic counseling is needed to provide risk estimates and inform patients and relatives. In case of an increased risk in a pregnancy prenatal echocardiography can be offered. However intra-familial phenotypic variability hampers prediction of age at onset and expected severity of feature [14]. In families where the genetic cause for NCCM has been identified, DNA testing of relatives and also prenatal DNA diagnostics can be performed [13].

The purpose of this paper is to discuss the clinical presentation, outcome, therapeutic options, natural history, and epidemiology of NCCM in pediatrics, including fetuses, neonates, infants, and children. Furthermore, we provide a simple classification of different forms of the disease. Finally, the differences between the pediatric population and the adult population are described.

## Historical perspective

The first case of NCCM was probably described by Bellet and Gouley in 1932 as a rare complex congenital myocardial anomaly [16]. They performed an autopsy on a newborn infant and found remains of the sinusoids of the embryonic heart and deep invaginations of the endocardium. However, in 1984, echocardiography allowed for the first diagnosis of an isolated NCCM, without concomitant structural CHD, in a living patient [2]. In 2006, an expert panel of the American Heart Association introduced NCCM as a distinct form of cardiomyopathy defined as a primary genetic heart disease characterized by a spongy myocardial appearance, predominantly involving the apex of the left ventricle, caused by an arrest in normal embryogenesis [1]. In contrast, the European Society of Cardiology (ESC) does not classify NCCM as a distinct cardiomyopathy [17]. Technological advancements in cardiac imaging in the last decades and increasing clinical awareness have allowed for more frequent recognition of NCCM.

## Epidemiology

The exact incidence of NCCM in children has not been established yet, due to the relatively recent classification as a distinct form of cardiomyopathy. Furthermore, consensus about diagnostic criteria has not yet been reached, leading to a delay in the diagnosis of NCCM or NCCM to be misdiagnosed as another form of CMP, such as DCM [18]. Differentiation of NCCM from DCM and NCCM with features of DCM could be important since treatment, prognosis, and rates of family occurrence may differ.

The estimated incidence of NCCM is 0.12 per 100.000 in children up to ten years of age and ≤ 0.81 per 100.000 in children up to one year of age [6]. NCCM is therefore considered as the third most common form of cardiomyopathy in children [6]. In addition, several smaller cohort studies in pediatric patients reported similar incidence rates. An Australian study reported an incidence of NCCM in 9.2% of all children diagnosed with a primary CMP under the age of ten between 1987 and 1996 [7]. Furthermore, according to the Pediatric Cardiomyopathy Registry (PCMR), a large register including 98 centers from the USA and Canada, 4.8% of all children diagnosed with any form of CMP had an isolated NCCM, over a period of 18 years [19]. Nonetheless, uniform diagnostic criteria and international registries are still warranted to determine the exact incidence and prevalence of NCCM.

## Pathophysiology

NCCM may involve abnormal myocardial embryogenesis. The heart forms as a simple tube with only two layers of cells, one epithelial and one endothelial, with a cardiac substance in between [20]. As the heart grows in mass, the cardiac substance is replaced by muscle tissue [20]. This newly formed muscle tissue is constructed in a sponge-like structure, enabling the cells to be oxygenated and receive nutrients through the blood flow in the endothelial outlined spaces, because of the absence of the coronary and sinusoidal circulation [11, 12, 21]. Normally, compaction of the myocardium takes place in weeks 5–8 of the fetal life, from basal to apical segments and from septal to lateral walls. In congenital NCCM, it is believed that genetic defects or epigenetic regulation of specific cardiac pathways cause an arrest in the normal process of myocardial compaction resulting in a myocardium consisting of two layers: one compact layer (epicardium) and one honeycomb-like structure with extensive ventricular trabeculation and deep intertrabecular recesses (endocardium) [11, 12, 20, 21].

Autopsy of a patient with isolated persisting myocardial sinusoids of both ventricles, an earlier terminology to define NCCM, shows the following features: hyperplastic trabeculae separated by labyrinthic spaces communicating with the ventricular cavity, an extended pericardial sac and thickening of the heart, mostly pronounced in the apical region [12]. Nonetheless, this bilayer structure of NCCM should be differentiated from trabeculae in the apex of the heart, which can be seen in healthy individuals. In the latter, the thickness of the trabecular layer does not usually exceed the compact layer in size, as in the case in NCCM [11, 12, 21].

NCCM predominantly affects the left ventricle, both in children and adults. To date, few cases of biventricular noncompaction in children are reported [22–27]. Furthermore, in the left ventricle, NCCM predominantly affects the apex. This can be explained by the fact that compaction of the heart proceeds from base to apex and form septal to lateral. However, the LV posterior wall may have hypertrabeculation as well.

## Pediatric NCCM phenotypes

Pediatric NCCM is typically a non-isolated phenotype that is often associated with concomitant features of other CMPs (mixed phenotype) or in the coexistence of one or more structural CHD(s), malformation syndrome, metabolic disorders, or NMD [13]. In contrast, the isolated form of NCCM is the most predominant phenotype in adult patients.

### NCCM with a mixed phenotype

**Fig. 1:**
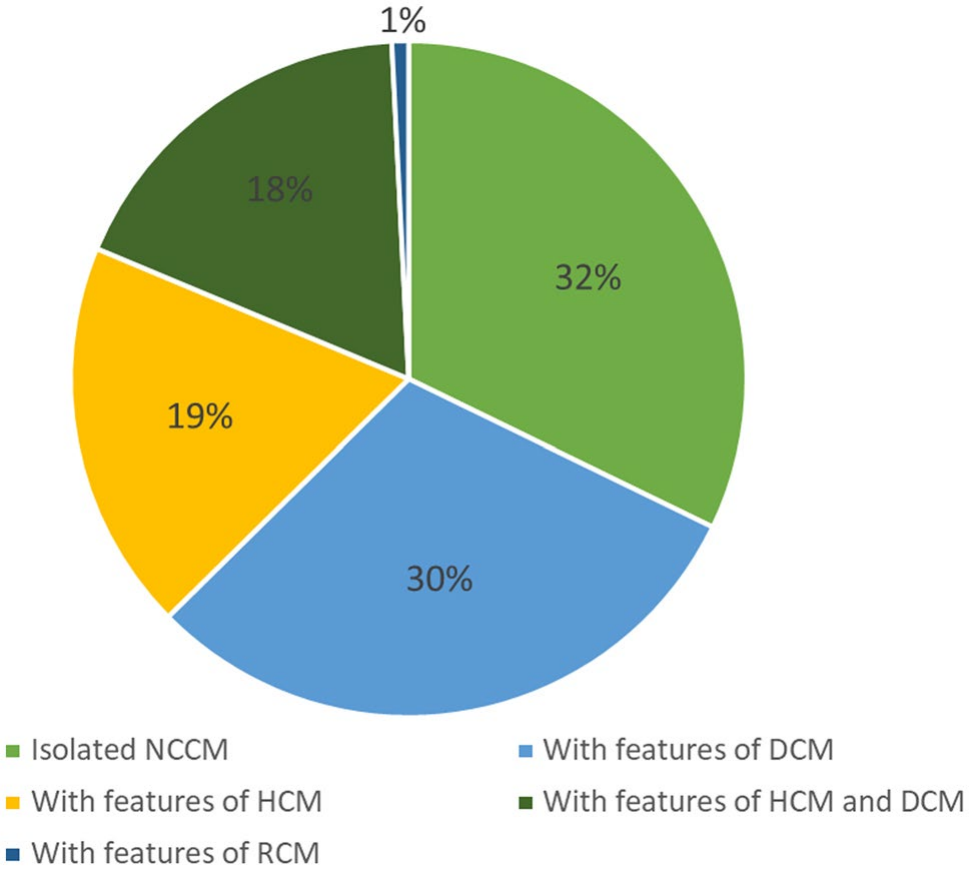
Concomitant features of other cardiomyopathies. Summarized data of the included cohort studies [7, 28–33]. DCM dilated cardiomyopathy, HCM hypertrophic cardiomyopathy, NCCM noncompaction cardiomyopathy, RCM restrictive cardiomyopathy

NCCM patients could present with features of another CMPs. Of the pediatric patients, 30.4% also present with DCM characteristics, 18.7% with HCM characteristics, and 17.9% with both DCM and HCM characteristics. Only 32.2% of the pediatric NCCM cases are without any features of another CMP (Fig. 1) [7, 28–33].

### NCCM with concomitant CHD

**Fig. 2:**
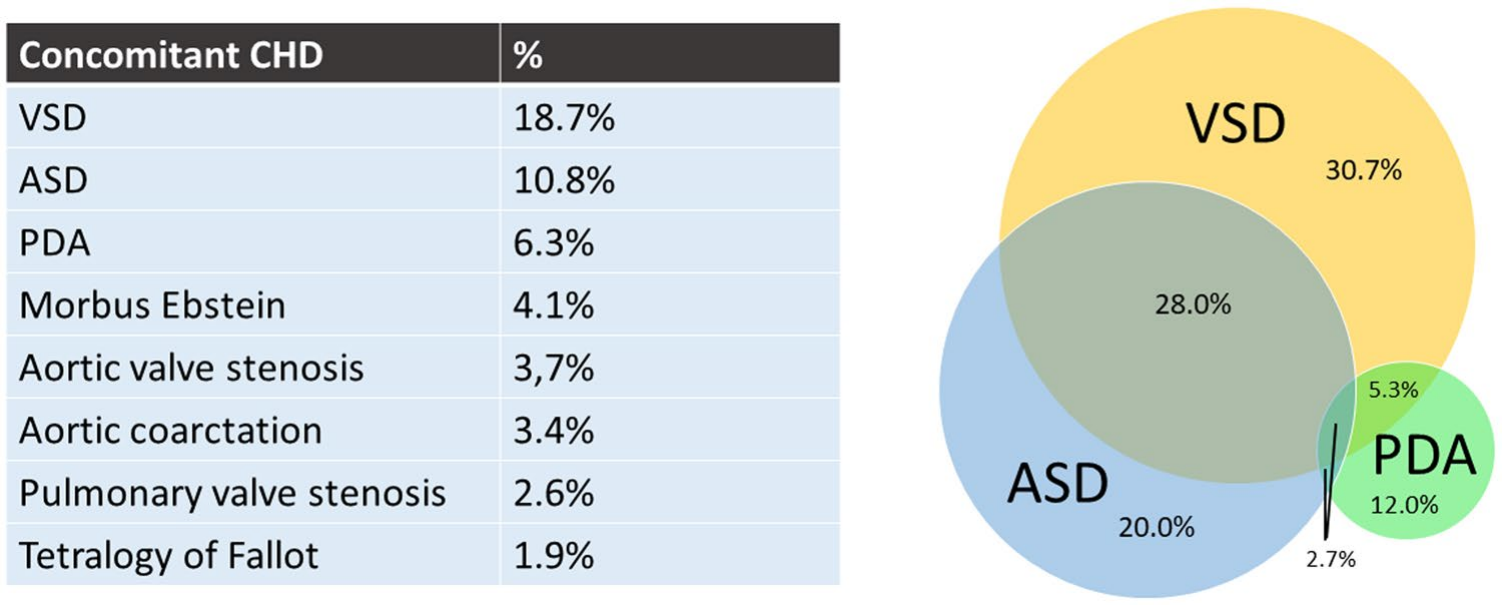
Combined percentages of concomitant congenital heart diseases reported in the included NCCM cohort studies [30–34, 36–39]. **b** Concomitant appearance of the three congenital heart defects mostly associated with noncompaction cardiomyopathy in children [30–34, 36–39]. ASD atrium septum defect, CHD congenital heart disease, PDA persistent ductus arteriosus, VSD ventricle septum defect

Pediatric NCCM has been reported with structural CHD [30–32, 34–39]. Initially, it was thought that NCCM only exists in the presence of CHD. The exact incidence of CHD in the NCCM population is unknown with incidence rates reported varying from 13 to 78% (Table 1). Ventricular septal defect (VSD; 18.7%), atrium septum defect (ASD; 10.8%), persistent ductus arteriosus (PDA; 6.3%), and Morbus Ebstein (4.1%) are the most reported forms of CHD in association with NCCM in the pediatric populations (Fig. 2a) [30–32, 34–39]. Figure 2b depicts the concomitant appearance of the three most common CHDs in the pediatric NCCM population.

**Table 1:** cohort studies on pediatric NCCM patients

	First author	Cohort period	n	Age	Male, *n* (%)	Method of diagnosis	Isolated NCCM or mixed CMP, *n* (%)		CHD, *n* (%)	Family history CMP, *n* (%)	Barth syndrome, *n* (%)	Follow-up	Arrhytmia, *n* (%)		Stroke/embolism	Death, *n* (%)	HTx, *n* (%)	Mode of death, *n* (%)	
Europe	Fazio (38)	1988–2005	21	Mean 4.9 years (range 21 days–27 years)	13 (62)	Echo Jenni criteria	Isolated NCCM Mixed CMP	9 (43) 12 (57)	11 (52)	8 (38)	0	Mean 7.8 years (range 1–18 years)	0			0	0		
	Lilje (39)	1999–2002	66	Med 4 years (range 0–21 years)	34 (52)	Echo; modified Chin (X/Y ratio ≤ 0.33)			41 (62)		0	Med 12 months (range 0–51 m)	Unspecified	13 (20)	Unspecified) thromboembolic events (14%)	5 (8)		Unspecified	
	van Waning	2005–2016	52	Genetic: med 5 (0–14), probably genetic: med 5 (0–13), sporadic: 8 (1–15)	27 (52)	Echo Jenni, CMR Petersen	Isolated NCCM	52	14 (27)	21 (40)	0	Med 60 months (IQR 18–113 months)	AF VT	5 (10) 1 (2)	Stroke (1) Peripheral thormbo-embolism (1)	4 (8)	4 (8)	Unspecified	
North America	Chin (21)	1983–1988	7	Med 5.5 years (range 1.5–14.5 years)	5 (71)	Echo Chin criteria			Excluded	4 (57)			VA WPW	5 (71) 1 (14)	2 cerebral emboli (29%)	2 (29)		iCVA HF	2 (29) 1 (14)
	Wald (29)	1988–2003	22	Mean 3.9 years (range 0–16 years)	9 (41)	Echo Jenni criteria	Isolated NCCM Mixed DCM Mixed RCM	4 (18) 17 (77) 1 (5)	Excluded	4 (18)	2 (9)	Med 3 years (range 0.1–16 years)	VA Afl WPW	2 (9) 1 (5) 1 (5)	0	3 (14)	2 (9)	SD HF	1 (5) 2 (9)
	Brescia (28)	1990–2009	242	Mean 7.2 years ± 6.9	145 (60)	Echo Jenni criteria	Isolated NCCM Mixed DCM Mixed HCM Mixed DCM/HCM Mixed RCM	63 (26) 46 (19) 65 (27) 68 (28) 0	Excluded	56 (23)	Excluded	Med 4.0 years (range 1.8–15.9 years)	VT AT rSVT AFl AJR AF WPW	42 (17) 14 (6) 19 (8) 4 (2) 2 (1) 1 (0,4) 20 (8)		31 (13)	13 (5)	SCD	15 (6)
	Zuckerman (34)	1993–2009	50	Med 0.3 years (range 1 day–21 years)	24 (48)	Echo	Mixed DCM	23 (46)	13 (26)		1 (2)					11 (22)	15 (30)	Unspecified	
	Tsai (37)	1999–2005	46	Med 0.44 years (0–18.5 years)	23 (50)	Echo			36 (78)	2 (4)	0	Mean 1.9 years ± 2.1	VT EA JR SVT WPW	2 (6) 2 (6) 2 (6) 2 (6) 3 (9)	0	9 (20)	0	Unspecified	
	Miller (9)	2009–2012	128	28% < 1 years, 21% 1–5 years, 24% 6–12 years, 24% 13–17 years, 3% 18–21 years	70 (55)	Echo Jenni or Stollberger criteria	Isolated NCCM Mixed CMP	76 (59) 52 (41)		33 (26)	1 (1)		ICD	6 (5)		2 (2)	1 (1)		
AU	Shi (7)	1987–1996	29	Med 0.3 years (IQR 0.1–1.3 years)	20 (69)	Echo Jenni criteria	Isolated NCCM Mixed DCM Mixed HCM Mixed RCM	1 (3) 27 (93) 0 1 (3)	Excluded	9 (31)	7 (24)	Med 6.8 years (IQR 0.7–24.0 years)	LBBB			14 (48)	6 (21)	SD	5 (17)
Asia	Ozkutlu (30)	1991–2002	12	med 1.5y (range 1 day–14 years)	11 (92)	Echo Chin criteria	Isolated NCCM Mixed DCM Mixed HCM Mixed RCM	9 (75) 2 (17) 1 (8) 0	7 (58)		0	Med 6 months (range 1 month–10 years)	SVT WPW Complete heart block	1 (8) 1 (8) 1 (8)	0	0	0		
	Wang (10)	1996–2014	108 (< 1 year)	Med 2.7 months	59 (55)	Echo Jenni criteria			Excluded	37 (34)	4 (2)	Med 4.9 years (range 1 day–22 years)	SVT LBTB VT AF AV-block SSS ICD PM WPW	5 (2) 7 (3) 11 (5) 4 (2) 8 (4) 5 (2) 2 (1) 3 (1) 14 (7)	5% systemic embolic event	14 (13)	5 (5)	HF VA SD iCVA LE During fetal period	12 (6) 2 (1) 4 (2) 1 (0.5) 2 (1) 1 (0.5)
			97 (1–15 years)	Med 7.3 years	51 (53)					35 (36)					5% systemic embolic event	9 (9)	4 (4)		
	Koh (36)	1999–2007	10	Med 2 years (range 7 days–12 years)	7 (70)	Echo Jenni criteria			3 (30)		1 (10)	Med 1.8 years (range 2 weeks–3 years)	VT SVT Suspected VA WPW	1 (10) 1 (10) 1 (10) 1 (10)	0	3 (30)	0	SD HF	2 (20) 1 (10)
	El-Menyar (31)	2000–2004	10	Mean 5.6 ± 3.5	3 (30)	Echo	Isolated NCCM Mixed HCM Mixed DCM/HCM	5 (50) 4 (40) 1 (10)	6 (60)	6 (60)		2–5 years	Atrial	3 (30)	0 Thromboembolic events	3 (30)		HF	3 (30)
	Ozgur (32)	2004–2009	29	Mean 4.8 years ± 4.6	16 (55)	Echo Jenni criteria	Isolated NCCM Mixed DCM Mixed HCM Mixed RCM	2 (7) 25 (87) 1 (3) 1 (3)	7 (24)	1 (3)	0	Mean 16 months (range 2 months–4 years)	VT	1 (3)	0	6 (21)	0	HF Pneumonia	4 (14) 2 (7)
	Ergul (35)	2006–2010	24	Mean 50 months ± 60	18 (75)	Echo and CMR			3 (13)			Mean 22 months ± 12			0	3 (13)		Unspecified	
	Tian (33)	2010–2016	41	Mean 14 years ± 4	28 (68)	CMR Petersen criteria	Isolated NCCM Mixed HCM	40 (98) 1 (2)	11 (27)	4 (10)		Mean 2.9 years ± 1.9	ICD	1 (2)	2% recurrent systemic embolism, no LV thrombosis	2 (5)	2 (5)	HF	2 (5)

*(A)JR* (accelerated) junctional rhythm, *AF* atrial fibbrilation, *AFl* atrial flutter, *AT* atrial tachycardia, *AU* Australia, *AV-block* atrioventricular block, *CHD* congenital heart disease, *CMP* cardiomyopathy, *DCM* dilated cardiomyopathy, *Fam* family, *EA* ectopic atrial rhythm, *HCM* hypertrophic cardiomyopathy, *HF* heart failure, *ICD* implantable cardiac defibrillator, *iCVA* ischemic cerbrovascular accident, *LBBB* left bundle branch block, *LE* lung embolism, *NCCM* noncompaction cardiomyopathy, *PM* pacemaker, *RCM* restrictive cardiomyopathy, *rSVT* reentrant supraventricular tachycardia, *SCA* sudden cardiac arrest, *S(C)D* sudden (cardiac) death, *SSS* sick sinus syndrome, *SVT* supraventricular tachycardia, *TE* thrombo-embolic, *VA* ventricular arrhythmia, *VT* ventricular tachycardia, *WPW* Wolff-Parkinson-White syndrome

### NCCM with concomitant NMD

Many NMDs eventually affect the cardiac muscle [40]. Consequently, NMDs are more frequently seen in cardiomyopathy populations, which also apply to the NCCM population [41]. The first described NMD patient with NCCM was an adult patient with Duchenne muscular dystrophy (DMD) [42]. The incidence of DMD in the pediatric NCCM population is unknown. However, the incidence of NCCM in the DMD population is reported near 20–25% [43, 44]. Little is known about the incidence of other neuromuscular disorders, like metabolic myopathy, Becker and Limb-Girdle syndrome [40], in the pediatric NCCM population and more research is warranted.

Another NMD associated with NCCM is Barth syndrome. This is syndrome will be discussed later.

### Fetal NCCM

If NCCM was an arrest in embryonic trabeculation, it should, in theory, be detectable at prenatal echocardiography. However, only a few cases of fetal NCCM diagnosis have been reported in the literature [14, 45–48]. Fetal hydrops was present in 33% of these cases and other structural cardiac abnormalities in 60% of patients [49]. The concomitant structural cardiac abnormalities most frequently reported in the fetal NCCM population were atrioventricular septal defect (33%), double-outlet right ventricle (26%), and left atrial isomerism (24%). The presence of Ebstein’s anomaly was not reported in this population [49].

The fetal NCCM population shows a trend towards a worse survival (37.5%, mean age 26 months). In addition, 9.1% died prenatally, 27.3% died after birth, and in 19.3% the parents decided to terminate the pregnancy. Also, 6.8% was lost to follow-up [49]. The seemingly worse survival in prenatal diagnosed NCCM patients could be due to the rareness of this new disease entity with publication bias, because prenatal echocardiography may only recognize the most severe cases [50].

## Genetics

NCCM, like most familial CMPs, is a genetic heterogeneous disease. Although this is a relatively young field of expertise, over 40 monogenetic and chromosomal defects are described in the overall NCCM population [51, 52]. Different inheritance patterns are described in NCCM [51–54] and the exact mechanism of how certain gene mutations can lead to NCCM is unknown. The same genetic defects are associated with different phenotypes, and the same genetic pathogenic variants can result in both overlapping and divergent cardiac manifestations, even within the same family. More importantly, a genotype-phenotype correlation has recently been established showing that specific genes may confer risk for overlapping cardiomyopathy phenotypes, like NCCM/DCM and NCCM/HCM within families [20, 52–55]. Furthermore, genetic defects are more often found in pediatric NCCM patients compared to the adult population [13]. However, since childhood NCCM is rare and routine genetic testing has not yet been widely applied, very limited data on the various (likely) pathogenic variants in pediatric NCCM exists. However, in our recent study we found a clear association with major adverse cardiac events and (probably) genetic NCCM, or familiar the genotypes, especially in children with multiple mutations in MYBPC3 (*p *= 0.006), along with presentation in neonates and very young children aged < 1 (HR of 2.1, *p *= 0.004) [13]. Future studies with comprehensive genetic testing could learn us more about the pathophysiology of NCCM, the incidence and severity of arrhythmias, risk of sudden cardiac death (SCD), and development of heart failure.

### Autosomal inheritance

In the overall NCCM population, various autosomal inherited genetic defects, mostly with dominant inheritance, were associated with NCCM, including pathogenic variants in sarcomere or cytoskeletal genes, and genes encoding ion channels. The sarcomere *MYH7* and *MYBPC3* genes are most frequently reported to cause NCCM (20–25% and 10%, respectively) [54]. Certain autosomal defects in other genes encoding proteins such as α-dystrobrevin, α-cardiac actin, and cardiac troponin T are also responsible for DCM and HCM. This implicates a possible similar molecular etiology to various cardiomyopathy phenotypes [8, 56–59]. Furthermore, DCM or HCM is often diagnosed in family members of NCCM patients. Interestingly, NCCM with features of DCM was associated with mutations in the tail of *MYH7* (*p* < 0.001) and NCCM with features of HCM was associated with *MYBPC3* (*p *< 0.001) [55].

In NCCM pediatric patients had concomitant congenital heart disease, the existing literature in these is scarce, but in a systemic review, most frequent mutations were in MYH7, MIB2, MKX2, NOTC1, NSD1, PTPN2, and a whole range of chromosomal defects [60].

### X-linked

The first pathogenic gene variant found to be responsible for NCCM was a genetic variant in the *TAZ* gene, on locus Xq28, with X-linked recessive inheritance. *TAZ* encodes for the protein tafazzin. Tafazzin is mostly expressed in cardiac and skeletal muscles and is involved in the metabolism of cardiolipin. Cardiolipin is important in maintaining the mitochondrial structure. Several other systemic myopathies, such as Barth syndrome, which is mostly accompanied by DCM, NCCM, or both, are also caused by a defect in this gene [61, 62].

Barth syndrome, or 3-methylglutaconic aciduria, is an X-linked metabolic disorder characterized by skeletal and cardiac myopathy, growth delay, and neutropenia in males [63]. It was first described by Harry Neustein in 1979 [64] and by a Dutch doctor named Peter Barth in 1983 [65]. Ninety percent of the boys with this extremely rare disease develop a form of cardiomyopathy [66]. In addition, 70% of these children were diagnosed with a CMP under 1 year of age, and all of them under the age of 5 [66]. In children with NCCM, the reported incidence rates of Barth syndrome range from 0 to 24%, with most studies reporting an incidence of 0% (Table 1) [7, 9, 10, 29, 30, 32, 34, 36–39]. The study that reported an incidence of 24% was a small study that included only 29 patients. Nevertheless, the wide incidence rate demonstrates this distinct cause for NCCM is frequently reported, even though the prevalence of Barth syndrome is low.

DMD, the first NMD in which NCCM concomitance was described, also follows an X-linked inheritance.

Interestingly, there is a possible difference in genetic pathophysiology between adult and pediatric patients. For example, genetic analyses performed on 25 adult patients showed no mutations in the *TAZ* gene. In addition, most of these adult patients presented with an autosomal dominant inheritance pattern, leading to the speculation that isolated NCCM in adults is mostly caused due to an autosomal dominant disorder, and therefore genetically distinct from pediatric X-linked causes [67].

### Mitochondrial

Various mitochondrial DNA (mtDNA), as well as nuclear genes involved in the oxidative phosphorylation, are linked to NCCM [52, 59]. Since Mt DNA diagnostics are not performed routinely for NCCM, the contribution of mtDNA pathogenic variants in NCCM may be larger [68].

## Clinical presentation

Children with non-isolated NCCM mostly present with the symptoms of their CHD [30].

**Fig. 3:**
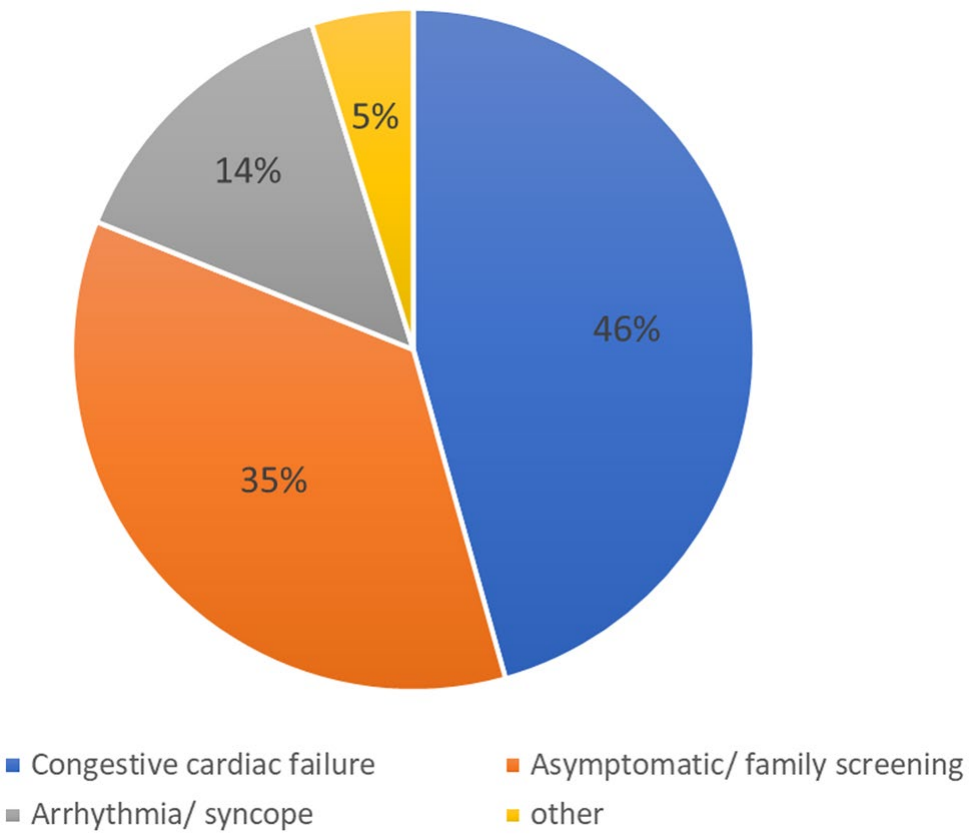
Initial presentation of noncompaction cardiomyopathy in children. Summarized data of the included cohort studies [7, 10, 21, 28, 29, 31–33, 35–38]

The most common clinical presentation of isolated NCCM in children is congestive cardiac failure (Fig. 3) [7, 10, 21, 28, 29, 31–33, 35–38]. However, patients with isolated NCCM can also be asymptomatic and diagnosed after an abnormal ECG, echocardiogram, chest X-ray, or simply after an abnormal cardiac exam. In contrast to adults, arrhythmia, chest pain, syncope, and (aborted) SCD are less common presenting symptoms in pediatric NCCM patients (Table 1).

Although the median age at presentation ranges between 0.3 and 5 years [7, 21, 30, 34, 36, 37, 39], several studies reported that children with NCCM are mostly diagnosed under the age of 1 year [9, 10, 28, 30, 34, 69].

In addition, pediatric NCCM patients with an HCM or DCM phenotype are significantly younger than NCCM patients without any concomitant features (9.3 years versus 0.4–0.6 years, *p* < 0.001) [19]. Furthermore, in comparison to pediatric patients with DCM, pediatric patients with NCCM listed for heart transplantation are significantly younger (3.1 ±4.3 years versus 6.9 ± 6.3 years, *p* < 0.0001) (70).

Interestingly, some pediatric patients show an improvement in their cardiac function after the initial diagnosis of NCCM. A brief period of myocardial recovery, prior to deterioration, proceeds. This so-called undulating phenotype may be an explanation for the presentation in adulthood [69]. For example, a national population-based study with a median follow-up of 24.7 years of all survivors found that normalization of left ventricular systolic function occurred in 8 of the 29 subjects (28%). Two of them later died suddenly and four of them showed reduced cardiac function at later follow-up [7].

Additionally, familial screening in NCCM has proven to be of significant value, since we found a combined incidence of 25.4% of the children diagnosed with NCCM to have a positive family history of NCCM or another form of CMP (Table 1) [7, 9, 10, 21, 28, 29, 31–33, 37, 38]. Furthermore, the NCCM phenotype of a patient may help predict CMP phenotypes and outcomes in relatives [55].

## Diagnostic

The diagnosis of NCCM is usually made through echocardiography or CMR according to the morphological criteria. Different echocardiographic criteria are proposed in the past 30 years to distinguish NCCM from physiological trabeculation and other forms of cardiomyopathy. The two most commonly used echocardiographic criteria are the Jenni criteria [4], the Chin criteria [21], and the Stöllberger criteria [71].

Most criteria, however, have poorly tested pathoanatomic correlation [4, 21, 72], and it is important to note that almost a fourth of the heart failure population fulfil one or more of these echocardiographic criteria. This suggests that the current echocardiographic criteria’s are not distinctive enough for NCCM.

**Fig. 4:**
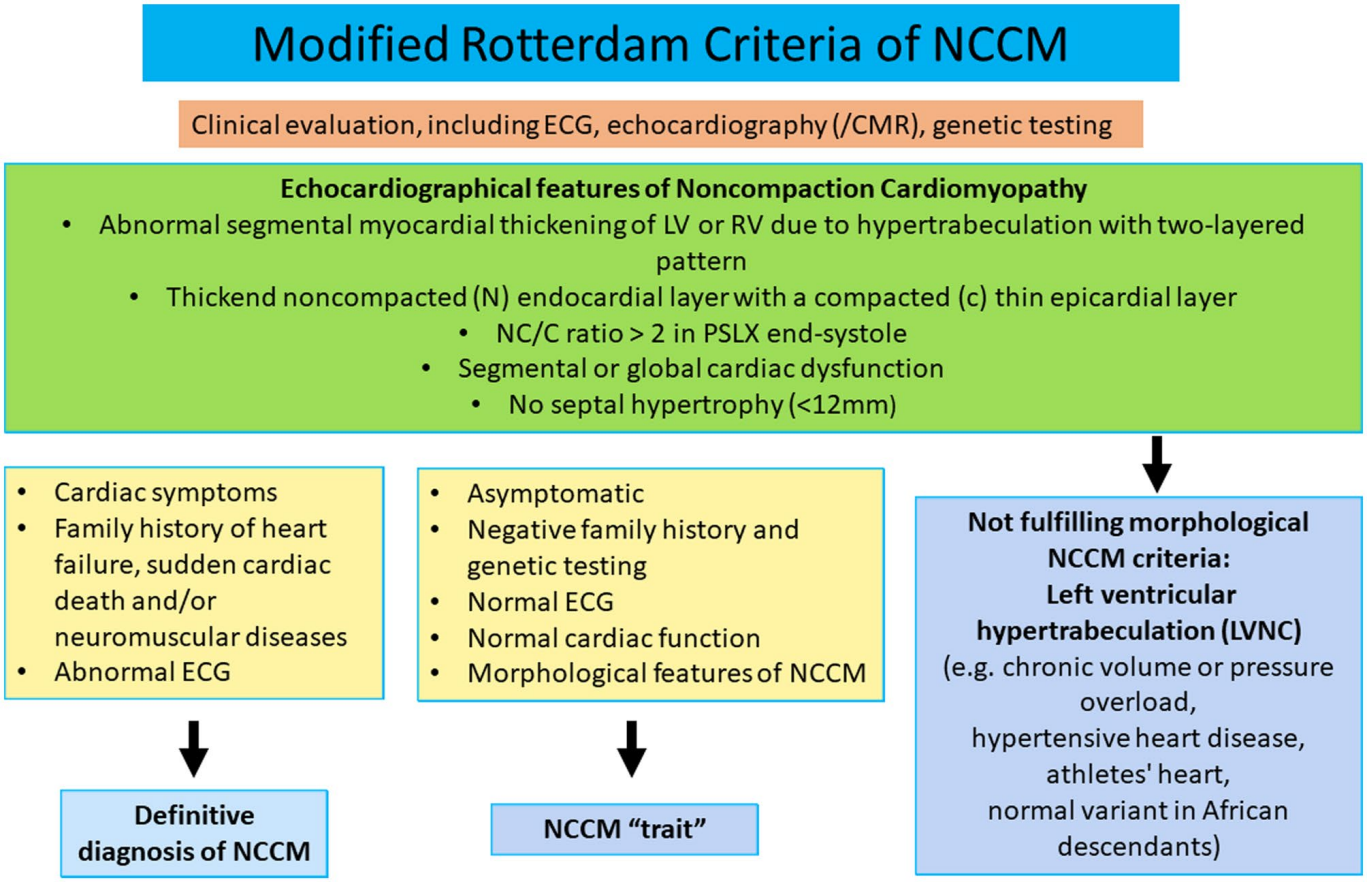
Diagnosing NCCM according to the Rotterdam criteria [18]. LVNC left ventricular noncompaction, NCCM noncompaction cardiomyopathy, NMD neuromuscular disease, PSLX parasternal long axis view

Therefore, our group proposed the Rotterdam criteria, combining both conventional trabeculation criteria (i.e., the “Jenni criteria”) and septal thickness (Fig. 4) [18]. These criteria differentiate between definite NCCM and a normal variant seen in athletes, African descendants, and long-standing hypertension [18]. Furthermore, differentiation between the fully asymptomatic patients with normal ECG, cardiac function, and genetics, i.e., not a disease in a narrow sense, is important because of the potential huge psychological, social, legal, and insurance consequences. Furthermore, eventual concomitant CHD or NMD should not be an exclusion criterion.

If uncertainty about the diagnosis of NCCM still exists, additional diagnostics, such as CMR, are warranted [73]. For CMR, the Petersen criteria are most frequently used in clinical practice [74]. NCCM can be distinguished from physiological trabeculation and other CMPs, such as HCM, through CMR, by a noncompacted layer to compact layer ratio of 2.3 (sensitivity of 86%, specificity of 99%) or a trabeculated LV mass ratio of > 20% (sensitivity and specificity of 94%) [74, 75]. These cohort studies were, however, rather small, and a larger, more recent study (*n* = 1480) showed that these features were present in 12.6% and 4.1% respectively of the healthy (adult) volunteers [76]. Interestingly, a recent study showed that meeting the Petersen criteria in ≥ 2 out of 3 long-axis views was an independent predictor for having a likely pathogenetic variant in adult NCCM patients who fulfilled the echocardiographic Jenni criteria [77].

Although echocardiography and CMR are frequently used to diagnose NCCM, no genetic, histopathological, or imaging diagnostic tool has been developed yet which can distinguish NCCM patients from physiologic hypertrabiculation of the left ventricle. The use of echocardiography and CMR has resulted in an exponential growth in the recognition of NCCM over the past 10 years. In a large multicenter study in North America, including 98 centers, 217 patients were diagnosed with NCCM between 2000 and 2009, while between 1990 and 1999, only 25 NCCM patients were identified [28]. This gain in “popularity” can be due to the raised awareness or the more flexible interpretation of the diagnostic criteria since various other disorders of the myocardium show high similarity [78].

Routine genetic testing is currently performed, albeit for now, there are no genes related specifically to NCCM and not to other cardiomyopathies [53, 54]. Furthermore, the same pathogenic gene variant can cause highly variable phenotypes [13]. However, if a family is known to be affected by a certain pathogenic gene variant, a molecular genetic analysis could be useful to identify other affected (asymptomatic) family members (cascade testing). Furthermore, genetic analyses should be considered, if the parents wish to have a child, to estimate the chance of re-occurrence. This is especially useful in case of (possible) childhood-onset NCCM, and in complex disorders presenting with NCCM, like in Barth syndrome. Genetic analysis yields the genetic cause in approximately 45% of childhood NCCM and may help determine the risk of recurrence and pathophysiology of NCCM [13].

## Natural history and prognosis

Reported outcomes in pediatric NCCM patients differ widely. An Australian study found that in 10 years after the diagnosis of NCCM with a dilated phenotype, half of the pediatric patients died or underwent HTx and that only 20% of the pediatric patients diagnosed with dilated NCCM were alive with normal cardiac function at 15-year follow-up [7]. A group in Toronto found a transplant-free survival rate after 3 years of 72% in a group of pediatric NCCM patients, of which 77.3% had cardiac dilatation [29]. In addition, a Japanese study reported poor outcomes (death/HTx) in only 11.1% of the pediatric patients after a median follow-up of 6 years (cardiac dilation in 33.3% of the patients) [79]. Colleagues in New York found a median transplant-free survival of 1.17 years after the presentation in pediatric NCCM patients (DCM phenotype in 46%) [34].

Differences in outcome can inter alia be explained by the composition of the cohort. Pediatric patients diagnosed with NCCM with mixed cardiomyopathy phenotypes have an increased risk for death or transplant compared (hazard ratio of 6.35) to NCCM patients without a mixed cardiomyopathy phenotype [19]. Furthermore, left ventricle dilatation is found to be an independent predictor of poor outcome (HTx/death) [34]. Likewise, a lower 5-year transplant-free survival was found in NCCM pediatric patients with a concomitant CMP in comparison to pediatric NCCM patients without concomitant CMP [28]. Time to death or HTx in children with dilated NCCM, however, did not differ from children with isolated DCM (*p* = 0.22). Composite end points at 5 years were 37% in the dilated NCCM group and 47% with only DCM [19].

Interestingly, patients with NCCM and Barth syndrome showed a trend towards better survival at 15 years than patients with NCCM alone (*n* = 7, 71% vs 36%, *p* = 0.08) [7]. The routine screening in families with Bart syndrome, and the consequential early diagnosis, may have contributed to this.

Furthermore, in the overall pediatric NCCM population, presentation in the first year of life is associated with increased mortality (HR 2.1, *p* = 0.02) [19, 28], and most deaths (90%) occur in the first year after the diagnosis of NCCM. This emphasizes the need for internationally accepted diagnostic criteria, which can be utilized to detect NCCM in an early stage of the disease.

## Management

No specific medical or surgical treatment strategy has yet been successfully introduced for the treatment of NCCM. Nevertheless, medical treatment with a beta-blocker, an angiotensin-converting enzyme inhibitor, and/or an angiotensin II receptor blocker might lead to favorable remodeling of the left ventricle [80]. No data exist for children with NCCM using newer therapies such as sacubitril/valsartan combination. Furthermore, children diagnosed with NCCM should be monitored closely for complications and deteriorations, and any arrhythmia should be treated according to clinical protocols. The effectiveness of ICD therapy needs to be evaluated in these patients. Additional effort should be made to determine if preventive anticoagulation or antiplatelet therapy is warranted in these children.

If, however, cardiac function deteriorates despite maximal medical treatment in children with end-stage heart failure due to NCCM, the only viable option for treatment is HTx. Overall, 4% of the children listed for HTx in North America were diagnosed with isolated NCCM. There was no difference in waiting list mortality or survival after HTx between pediatric NCCM patients and pediatric DCM patients. However, there was a decreased freedom of infection in NCCM patients compared to DCM patients under 1 year of age. Known NCCM-immune deficiencies may have contributed to this fact. However, refusing HTx to pediatric NCCM patients is not grounded based on the diagnosis alone, since graft rejection and mortality after HTx is similar in NCCM and DCM patients [70]. Experience with mechanical circulatory support in pediatric patients with NCCM is scarce and limited to single cases [81–83]. Though it is feasible, this therapy is reserved for only NCCM patients with single, predominantly left, ventricular failure. Larger studies are warranted to depict the outcomes of pediatric NCCM patients treated with mechanical circulatory support.

## Complications

NCCM is often associated with various tachyarrhythmias, with incidence rates reported between 0 and 43% (Table 1) [21, 28–32, 36–39]. The presence of arrhythmia is a known risk factor for several morbidities and mortality in children with NCCM [28]. Therefore, more research on the exact incidence and severity of the various forms of arrhythmias and management of complications of pediatric NCCM patients is warranted.

Another association frequently made with NCCM is the occurrence of stroke and other thromboembolic events. It is believed that blood clots are likely to form in the honeycomb-like structure of the myocardium, causing regions of blood stasis in NCCM patients. Reported incidence of thromboembolism in NCCM children ranges from 0 to 29% (Table 1) [10, 21, 29–33, 35–37, 39]. The occurrence of stroke was only found in one cohort study (2 patients affected, 29%) [21]. In children with NCCM on the HTx waiting list, stroke occurred in 4% [70]. Interestingly, most studies (7/11) on embolisms in NCCM children report an incidence of 0% (Table 1) [29–32, 35–37].

In adults, however, the incidence of embolism ranges from 5 to 38% and is therefore one of the most feared complications in NCCM patients. Consequently, antithrombotic prophylaxis is prescribed by some medical centers [84].

More research on the exact incidence of thromboembolic events in NCCM children is warranted to evaluate if anticoagulation or antiplatelet therapy in these children is necessary and justified.

## Future perspective

Considering the rareness of the disease, there is a need for an international consensus on the diagnostic criteria and classification of NCCM as a distinct form of cardiomyopathy. Echocardiography and other minimal invasive diagnostics should be further studied to enable physicians to diagnose NCCM through simple, non-invasive and widely available tools. A joint international effort with attention to the different subtypes of NCCM would be beneficial for the cardiac society. Hopefully, the near future will witness, (inter)national, multicenter collaborations to collect cases in a registry in order to have data from which the outcome and impact of management strategies in these patients can be evaluated, to improve clinical management of patients and their families.

## Conclusion

Childhood NCCM is a novel, yet rare clinical entity with heterogeneous phenotypes, clinical presentation, and potentially fatal complications. Clinical presentation varies widely from asymptomatic to congestive heart failure and sudden cardiac arrest. The diagnosis of NCCM, performed through echocardiography or CMR, would less frequently be missed through raised awareness and operator training. Systematic DNA testing is encouraged since in nearly half cases a genetic cause can be identified, and genetics may predict clinical outcome. There is a need for an international consensus on the classification of NCCM as distinct cardiomyopathy and its diagnostic criteria. Finally, efforts are needed to collect cases in a registry in order to recognize outcome patterns and evaluate the impact of management strategies in these patients.

## Clinical perspectives

- Noncompaction cardiomyopathy (NCCM) is a novel, yet rare clinical entity, but nevertheless the third most common cardiomyopathy in childhood and is associated with congestive heart failure, arrhythmias and/or thromboembolic events.
- This state-of-the-art review provides an overview of pediatric NCCM including the epidemiology, natural history, genetics, clinical presentation, prognosis, and therapeutic options of NCCM in pediatric patients.
- There is an urgent need for an international consensus on the classification of NCCM, the diagnostic criteria, and use of genetic testing in the daily clinical practice.
- Finally, an effort should be made to collect cases in a multicenter, international registry in order to evaluate the outcome and impact of management strategies in these patients.

## Abbreviations

CMR: Cardiac magnetic resonance imaging
Htx: Heart transplantation
IQR: Interquartile range
LV: Left ventricle
Med: Median
